# Eltrombopag Inhibits Metastasis in Breast Carcinoma by Targeting HuR Protein

**DOI:** 10.3390/ijms24043164

**Published:** 2023-02-05

**Authors:** Yao Chen, Rui Zhang, Liuqing Yang, Pei Zhang, Feiyun Wang, Guoqiang Lin, Jiange Zhang, Yuying Zhu

**Affiliations:** 1 The Research Center of Chiral Drugs, Innovation Research Institute of Traditional Chinese Medicine, Shanghai University of Traditional Chinese Medicine, Shanghai 201203, China; 2 Shanghai Frontiers Science Center for Traditional Chinese Medicine Chemical Biology, Innovation Research Institute of Traditional Chinese Medicine, Shanghai University of Traditional Chinese Medicine, Shanghai 201203, China; 3 Shanghai Institute of Traditional Chinese Medicine, Shanghai 201203, China

**Keywords:** eltrombopag (ELB), HuR, macrophage, breast cancer, lung metastasis, lymph node metastasis

## Abstract

Eltrombopag is a small molecule TPO-R agonist that has been shown in our previous studies to inhibit tumor growth by targeting Human antigen R (HuR) protein. HuR protein not only regulates the mRNA stability of tumor growth-related genes, but it also regulates the mRNA stability of a variety of cancer metastasis-related genes, such as *Snail*, *Cox-2*, and *Vegf-c*. However, the role and mechanisms of eltrombopag in breast cancer metastasis have not been fully investigated. The purpose of this study was to investigate whether eltrombopag can inhibit breast cancer metastasis by targeting HuR. Our study first found that eltrombopag can destroy HuR-AU-rich element (ARE) complexes at the molecular level. Secondly, eltrombopag was found to suppress 4T1 cell migration and invasion and inhibit macrophage-mediated lymphangiogenesis at the cellular level. In addition, eltrombopag exerted inhibitory effects on lung and lymph node metastasis in animal tumor metastasis models. Finally, it was verified that eltrombopag inhibited the expressions of *Snail*, *Cox-2*, and *Vegf-c* in 4T1 cells and *Vegf-c* in RAW264.7 cells by targeting HuR. In conclusion, eltrombopag displayed antimetastatic activity in breast cancer in an HuR dependent manner, which may provide a novel application for eltrombopag, hinting at the multiple effects of HuR inhibitors in cancer therapy.

## 1. Introduction

Breast cancer is the most commonly diagnosed cancer worldwide (over 2.26 million cases). It is a disease with high fatality rate and poor prognosis, and it is characterized by distant metastasis [1]. Triple-negative breast cancer, in particular, is the most typical type of breast cancer, accounting for 15–20% of breast cancer metastases, and there is no effective therapeutic target. Currently, some FDA-approved drugs have been applied to treat breast cancer metastases, such as chemotherapy agents, but they have also shown the limitations of drug resistance [2]. Therefore, the treatment of breast cancer metastases still remains a challenge. It is urgent to explore new therapeutic strategies for breast cancer metastasis.

Epithelial–mesenchymal transition (EMT) is considered to be a vital step in invasion, which is characterized by loss of epithelial cells markers (e.g., E-cadherin) and the upregulation of mesenchymal cell markers (e.g., vimentin and N-cadherin) [3]. *Snail* is the most important transcriptional repressor of E-cadherin and plays a key role in EMT, which is responsible for the invasion and metastasis of tumors [4,5]. Cyclooxygenase-2 (*Cox-2*) is an inducible enzyme associated with prostaglandin synthesis. Although *Cox-2* is known to play a key role in inflammation, it has been well established that *Cox-2* promotes carcinogenesis and is associated with tumor growth, angiogenesis, and metastasis [6,7,8]. Vascular endothelial growth factor C (*Vegf-c*), a member of the VEGF family, promotes the proliferation and migration of lymphatic endothelial cells (LECs) by activating VEGF receptor-3 (VEGFR-3), which is expressed in lymphatic endothelial cells (LECs) [9]. Studies have shown that *Vegf-c* promotes tumor lymphangiogenesis and ultimately leads to multiple cancer metastases [10,11]. Although cancer cells can produce *Vegf-c* and other factors to increase cancer metastasis, macrophages and other immune cells are also important sources of *Vegf-c* in tumor microenvironments [12,13]. More importantly, clinical studies on human tumor samples have shown that the high expression of *Vegf-c* is related to metastasis and predicts poor prognosis [14,15,16]. Thus, *Snail*, *Cox-2*, *Vegf-c*, and macrophages are closely related to tumor invasion and metastasis.

Human antigen R (HuR), as an RNA-binding protein, stabilizes the target mRNA and increases translation by binding to the AU-rich element (ARE) of the 3′ untranslated region (3′ UTR) of mRNA [17]. HuR is abnormally overexpressed in the cytoplasm of cancers, such as breast cancer, colon cancer, and prostate cancer, and regulates tumors by the post-transcriptional regulation of the mRNA stability of genes related to tumor growth, angiogenesis, metastasis, and progression [18,19,20].

The post-transcriptional regulation of *Snail*, *Cox-2*, *Vegf-c,* and other factors may be affected by the HuR protein. This is due to the abundant ARE in the 3′ UTR of their mRNAs, which easily binds to the HuR protein. The mechanism by which the HuR protein regulates *Snail* mRNA stability in a UDP-glucose-dependent manner during lung cancer metastasis was recently revealed [21]. In addition, HuR stabilizes *Cox-2* mRNA and is associated with tumor progression in colon cancer and breast cancer [22,23]. Similarly, the increased binding of HuR to *Vegf-c* mRNA has been shown to promote lymphangiogenesis and lymph node metastasis in esophageal squamous cell carcinoma and non-small-cell lung cancer [24,25].

Multiple HuR inhibitors have been reported to inhibit tumor cell migration, including KH-3, MS-444, and N-Benzylcantharidinamide [26,27,28]. Among them, KH-3 was shown to inhibit lung metastasis of breast cancer in vivo by suppressing the HuR-FOXQ mRNA interaction [29]. Therefore, HuR might be an efficient target for tumor metastasis, and more in vivo data and HuR-dependent mechanisms are needed to support this hypothesis.

Eltrombopag is a small molecule thrombopoietin receptor (TPO-R) agonist that has been used for treating patients with chronic immune thrombocytopenic purpura (ITP) [30]. Our previous research identified eltrombopag as a novel HuR inhibitor that plays an anti-breast cancer growth role by targeting HuR to regulate tumor angiogenesis [31]. In this study, we investigated the therapeutic effect of eltrombopag in breast cancer metastasis by targeting HuR. We first identified the disruptive effect of eltrombopag on the interaction between the HuR protein and the ARE sequences of tumor metastases-associated genes (*Snail*, *Cox-2*, and *Vegf-c*). Then, breast cancer cells (4T1) were used as research objects to explore the inhibitory effects of eltrombopag on cell migration and invasion and the mechanism of targeting HuR. Furthermore, we investigated the inhibitory effect of eltrombopag on lymphangiogenesis mediated by macrophages (RAW264.7), thereby elucidating its important role in tumor metastasis and the mechanism of eltrombopag’s dependence on HuR. We also evaluated the effect of eltrombopag on breast cancer metastasis in vivo. Our work reveals that eltrombopag suppressed breast cancer metastasis by acting on the HuR protein, which suggests that the HuR protein is a potential target for breast cancer metastasis inhibition.

## 2. Results

### 2.1. Eltrombopag Blocks the Binding of HuR Protein to ARE Sequences of Snail, Cox-2 and Vegf-c mRNAs

**Figure 1:**
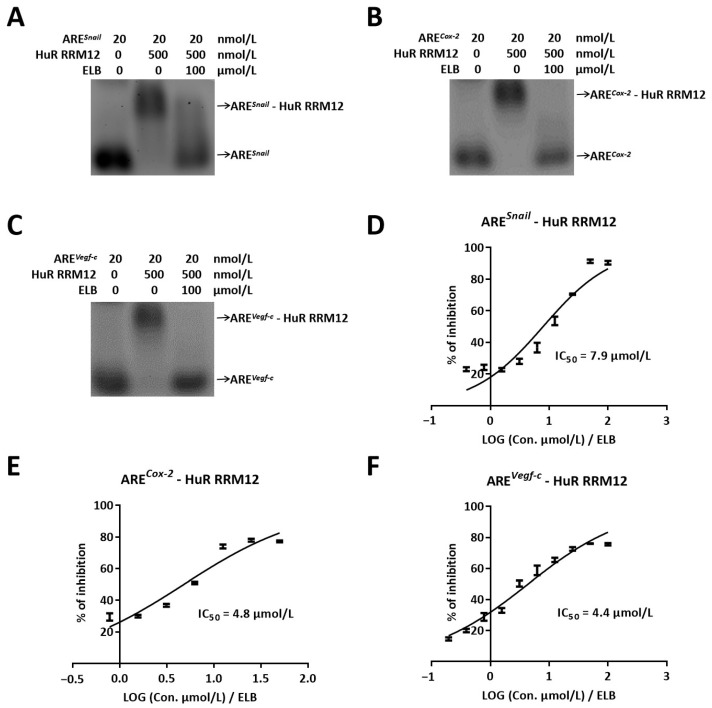
Eltrombopag interrupted the interaction between HuR RRM12 domain and ARE sequences (ARE*^Snail^*, ARE*^Cox-2^*, and ARE*^Vegf-c^*). (**A**–**C**) The inhibitory effect of eltrombopag on the binding of HuR RRM12 and ARE*^Snail^*, ARE*^Cox-2^*, and ARE*^Vegf-c^* by EMSA method. (**D**–**F**) The IC_50_ of eltrombopag disrupts binding of HuR RRM12 to ARE (*Snail*, *Cox-2,* and *Vegf-c*) by FP method. *n* = 3.

Our previous work identified the HuR protein as a novel target for the anti-breast cancer activity of eltrombopag and demonstrated that the binding site of eltrombopag is within the RRM12 domain of the HuR protein. *Snail*, *Cox-2*, and *Vegf-c* are important genes associated with tumor metastasis, and their mRNA stability is regulated by HuR. Thus, we first investigated the inhibitory effect of eltrombopag on the binding of the HuR protein to ARE sequences of *Snail*, *Cox-2*, and *Vegf-c* mRNAs. By EMSA, we observed that eltrombopag could completely destroy the interactions of RRM12-ARE*^Snail^* (Figure 1A), RRM12-ARE*^Cox-2^* (Figure 1B), and RRM12-ARE*^Vegf-c^* (Figure 1C) at the concentration of 100 μmol/L. In addition, the inhibitory rate of eltrombopag on the interaction between HuR RRM12 and AREs was analyzed by the FP method. The IC_50_ of eltrombopag for the RRM12-ARE*^Snail^* interaction was 7.9 μmol/L (Figure 1D) and 4.8 μmol/L for the RRM12-ARE*^Cox-2^* interaction (Figure 1E). The IC_50_ for the RRM12-ARE*^Vegf-c^* interaction was 4.4 μmol/L (Figure 1F). Therefore, in vitro studies have shown that eltrombopag has the ability to inhibit the binding of the AREs of tumor metastasis-related genes (*Snail*, *Cox-2*, and *Vegf-c*) to HuR.

### 2.2. Eltrombopag Inhibits the Migration and Invasion of Breast Cancer Cells

**Figure 2:**
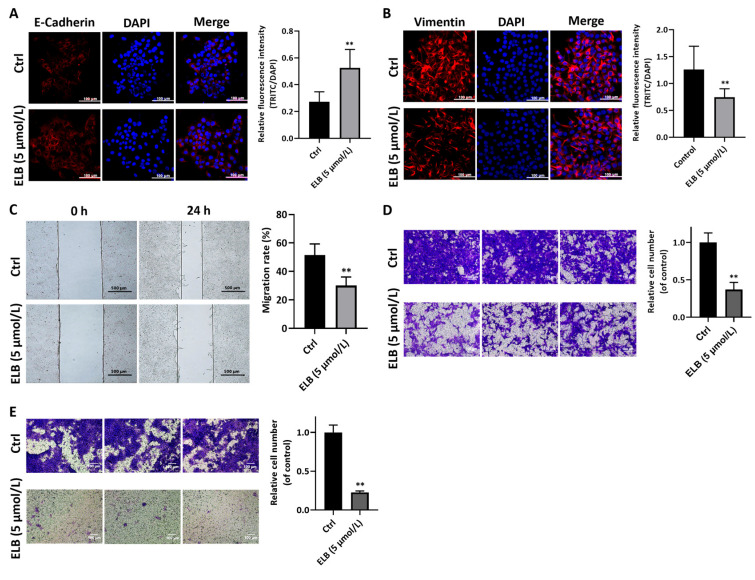
The role of eltrombopag in migratory and invasive of 4T1 cells. (**A**) Impact of eltrombopag to expression of E-cadherin protein in 4T1 cells, representative images and quantitative data of immunofluorescence experiments are shown (Scale bar: 100 μm). (**B**) Impact of eltrombopag to expression of vimentin protein in 4T1 cells, representative images and quantitative data of immunofluorescence experiments are shown (Scale bar: 100 μm). (**C**) Scratch wound assay was used to determine the migration effect of eltrombopag on 4T1 cells, representative images and quantitative data are shown (Scale bar: 500 μm). (**D**) Transwell migration experiment was used to determine the vertical migration effect of eltrombopag on 4T1 cells, representative images and quantitative data are shown (Scale bar: 100 μm). (**E**) Transwell invasion assay was used to investigate the inhibitory effect of eltrombopag on the invasion of 4T1 cells, representative images and quantitative analyses are shown (Scale bar: 100 μm). Mean ± SD. *n* = 3. ** *p* < 0.01 vs. control group (*t*-test).

**Figure 3:**
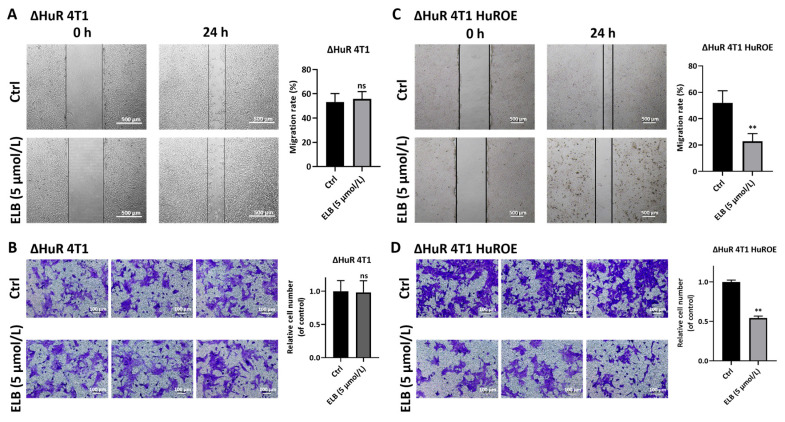
The role of eltrombopag in migratory of HuR-knockout 4T1 cells (ΔHuR 4T1) and HuR-rescued ΔHuR 4T1 cells (ΔHuR 4T1 HuROE). (**A**) Wound healing assay was used to determine the migration of eltrombopag on ΔHuR 4T1 cells, representative images and quantitative data are shown (Scale bar: 500 μm). (**B**) Transwell migration experiment was used to determine the vertical migration effect of eltrombopag on ΔHuR 4T1 cells, representative images and quantitative data are shown (Scale bar: 100 μm). (**C**) Wound healing assay was used to determine the migration of eltrombopag on ΔHuR 4T1 HuROE cells, representative images and quantitative data are shown (Scale bar: 500 μm). (**D**) Transwell migration experiment was used to determine the vertical migration effect of eltrombopag on ΔHuR 4T1 HuROE cells, representative images and quantitative data are shown (Scale bar: 100 μm). Mean ± SD. *n* = 3. ** *p* < 0.01 vs. control group (*t*-test). ns means no significant difference.

Eltrombopag showed an inhibition effect on multiple breast cancer cell lines, including 4T1, MDA-MB-468, and MCF-7 (Figure S1). As 4T1 cells showed a high invasion ability, we further study eltrombopag’s inhibition effect with 4T1 cells. In this part, we first detected two EMT markers impacted by eltrombopag in 4T1 cells. E-cadherin was significantly elevated by 5 μmol/L eltrombopag (Figure 2A, *p* < 0.01), and vimentin was significantly inhibited by 5 μmol/L eltrombopag (Figure 2B, *p* < 0.01). Then, we assessed the inhibitory effect of eltrombopag on the migration and invasion function of 4T1 cells by using a scratch wound assay and transwell assay. In the scratch wound experiment, we found that eltrombopag (5 μmol/L) significantly reduced the migration of the 4T1 cells compared to the control group (Figure 2C, *p* < 0.01). Through the transwell migration experiment (Figure 2D), we demonstrated that eltrombopag (5 μmol/L) could remarkably reduce the vertical migration abilities of 4T1 cells in vitro (*p* < 0.01). Similar results were also obtained by the transwell invasion assay of 4T1 cells (Figure 2E, *p* < 0.01). Finally, we further detected the invasion and migration function of ΔHuR 4T1 (HuR-knockout cell line) cells and ΔHuR 4T1 HuROE (HuR expressed in HuR-knockout cell line) cells when treated with eltrombopag (5 μmol/L). As shown in Figure 3, 5 μmol/L of eltrombopag could not inhibit the cell migration of ΔHuR 4T1 in the wound-healing experiment (Figure 3A, *p* > 0.05) and the transwell migration experiment (Figure 3B, *p* > 0.05). However, when plasmid with the HuR gene was transfected into ΔHuR 4T1 cells, the inhibitory effect of eltrombopag on cell migration was recovered (Figure 3C, *p* < 0.01, and Figure 3D, *p* < 0.01). Therefore, eltrombopag inhibited the migration and invasion function of 4T1 cells in an HuR-dependent manner.

### 2.3. Eltrombopag Inhibits the Expression of Genes Associated with Tumor Metastasis in 4T1 Cells by Targeting HuR Protein

**Figure 4:**
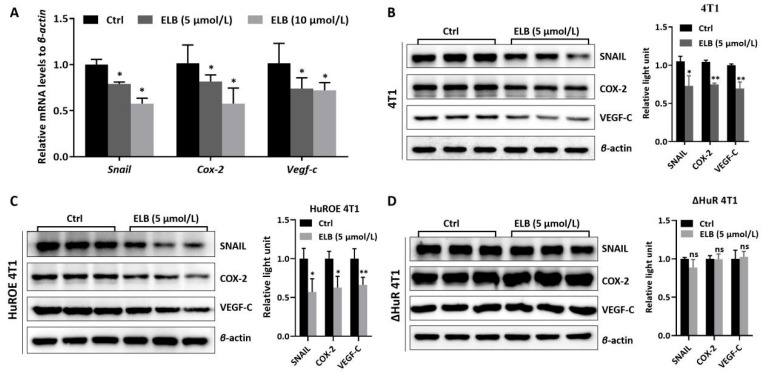
Impact of eltrombopag on the expression of *Snail*, *Cox-2,* and *Vegf-c* in 4T1, HuROE 4T1, or ΔHuR 4T1 cells. (**A**) The mRNA levels of *Snail*, *Cox-2,* and *Vegf-c* in 4T1 cells were tested by qRT-PCR. (**B**) The protein expression of *Snail*, *Cox-2,* and *Vegf-c* was detected in 4T1 cells via Western blot, photographs, and quantification. (**C**) The protein expression of *Snail*, *Cox-2*, and *Vegf-c* was detected in HuROE 4T1 cells via Western blot, photographs, and quantification. (**D**) The protein expression of *Snail*, *Cox-2,* and *Vegf-c* was detected in ΔHuR 4T1 cells via Western blot, photographs, and quantification. Mean ± SD. *n* = 3. * *p* < 0.05 (*t*-test), ** *p* < 0.01 (*t*-test), ns means no significant difference.

We further verified whether the inhibitory effect of eltrombopag on 4T1 cell migration and invasion was achieved by targeting HuR and reducing the mRNA stability of tumor metastasis-related genes (*Snail*, *Cox-2*, and *Vegf-c*). First, *Snail*, *Cox-2*, and *Vegf-c* mRNA levels in 4T1 cells were detected by qRT-PCR. As shown in Figure 4A, eltrombopag (5 μmol/L and 10 μmol/L) significantly reduced *Snail*, *Cox-2*, and *Vegf-c* mRNA levels in 4T1 cells compared to the control group (*p* < 0.05). Then, the HuR overexpressed cell line, HuROE 4T1, and the HuR-knockout cell line, ΔHuR 4T1, was constructed. The effects of eltrombopag on *Snail*, *Cox-2*, and *Vegf-c* protein levels in 4T1, HuROE 4T1, and ΔHuR 4T1 cells were detected by Western blot. The results show that eltrombopag (5 μmol/L) greatly down-regulated the protein expressions of *Snail* (*p* < 0.05), *Cox-2* (*p* < 0.01), and *Vegf-c* (*p* < 0.01) in the 4T1 cells (Figure 4B) and in the HuROE 4T1 cells (Figure 4C, *Snail*, *p* < 0.05; *Cox-2*, *p* < 0.05; *Vegf-c*, *p* < 0.01). Nevertheless, there were no significant differences in the expression of these proteins in the ΔHuR 4T1 cells compared to the control group (Figure 4D, *p* > 0.05).

**Figure 5:**
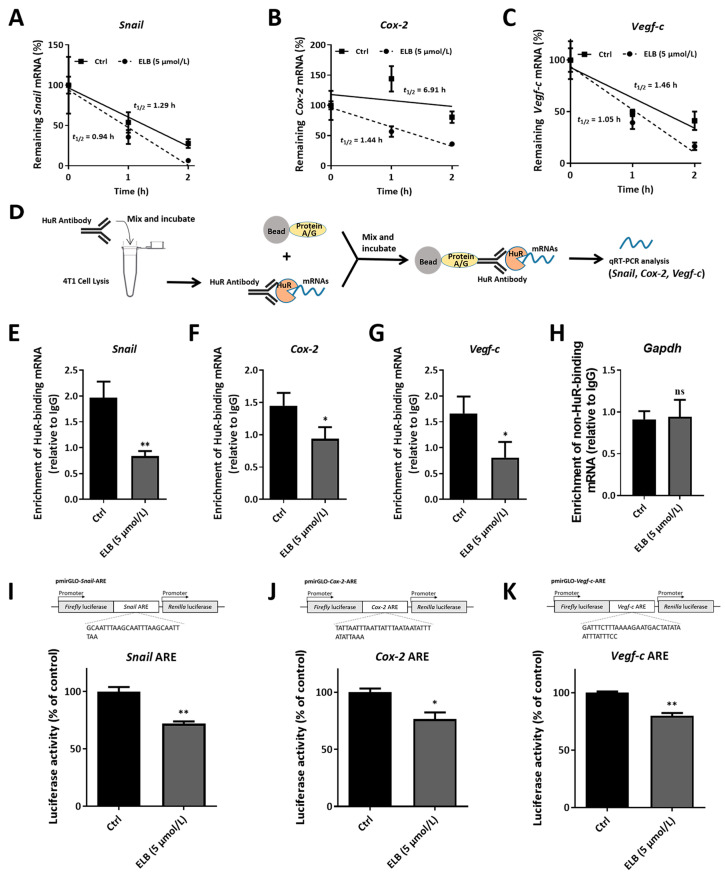
Eltrombopag regulated the function of the HuR protein in 4T1 cells. (**A**–**C**) The mRNA stability of *Snail*, *Cox-2,* and *Vegf-c* in 4T1 cells was impacted by eltrombopag. (**D**) Schematic diagram of the RIP analysis. (**E**–**H**) HuR-binding mRNA (*Snail*, *Cox-2,* and *Vegf-c*) and non-HuR-binding mRNA (*Gapdh*) levels in 4T1 cells influenced by eltrombopag detected with RIP. (**I**–**K**) Luciferase activity mediated by *Snail*-ARE, *Cox-2*-ARE, and *Vegf-c*-ARE in 4T1 cells impacted by eltrombopag. Mean ± SD. *n* = 3. * *p* < 0.05 (*t*-test), ** *p* < 0.01 (*t*-test), ns means no significant difference.

The mRNA stability of *Snail*, *Cox-2*, and *Vegf-c* in 4T1 cells was then detected. The data show that the *Snail*, *Cox-2*, and *Vegf-c* mRNA half-life time were all declined in 4T1 cells when exposed to eltrombopag (Figure 5A–C). Subsequently, RIP analysis was used to detect the binding of HuR to the mRNAs of *Snail*, *Cox-2*, and *Vegf-c* in 4T1 cells. As shown in Figure 5, *Snail* (Figure 5E, *p* < 0.01), *Cox-2* (Figure 5F, *p* < 0.05), and *Vegf-c* (Figure 5G, *p* < 0.05) mRNA levels were evidently decreased in HuR immunoprecipitation complexes compared to IgG immunoprecipitation complexes in 4T1 cells by eltrombopag, while the non-HuR-binding mRNA *Gapdh* was not affected by eltrombopag (Figure 5H, *p* > 0.05). We also designed a luciferase assay to test the ARE-dependent gene expression in 4T1 cells, in which the ARE sequence was inserted into the 3′-UTR of the firefly luciferase gene in the pmirGLO vector and transfected into 4T1 cells. The results show that eltrombopag significantly inhibited the activity of firefly luciferase (Figure 5I–K, *Snail* ARE, *p* < 0.01; *Cox-2* ARE, *p* < 0.05; *Vegf-c* ARE, *p* < 0.01). Taken together, our results suggest that eltrombopag affected the migration and invasion of breast cancer cells by targeting the HuR protein and inhibiting its binding to the mRNA of tumor metastasis-related genes, thereby inhibiting the expressions of related genes.

### 2.4. Eltrombopag Inhibits Macrophage-Mediated Lymphangiogensis by Inhibiting HuR Protein Function

**Figure 6:**
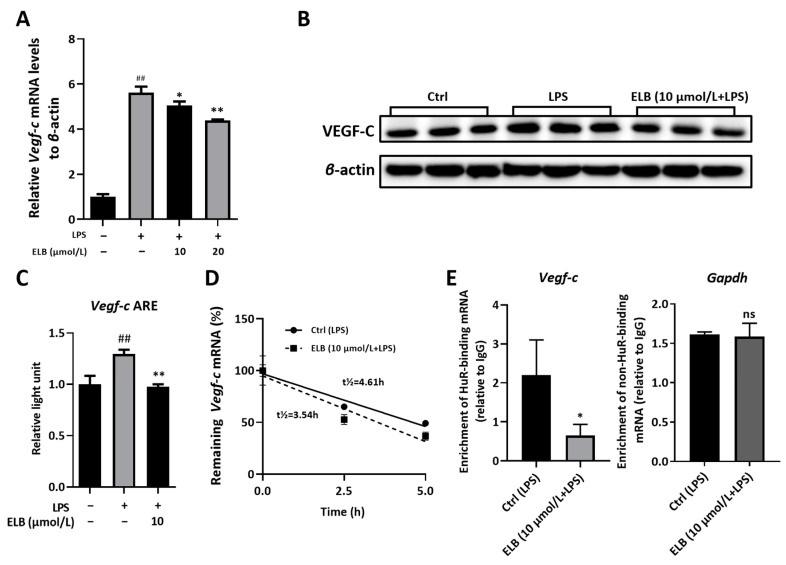
Eltrombopag regulated lymphangiogenesis-related gene in RAW264.7 cells by targeting HuR. (**A**) Relative mRNA level of *Vegf-c* in RAW264.7 cells impacted by eltrombopag. (**B**,**C**) Relative protein expression of *Vegf-c* in RAW264.7 cells impacted by eltrombopag measured with Western blot, representative images and quantitative analyses. (**D**) mRNA stability of *Vegf-c* in RAW264.7 cells impacted by eltrombopag. (**E**) RIP analysis detected the enrichment of HuR-binding mRNA (*Vegf-c*) and non-HuR-binding mRNA (*Gapdh*) in RAW264.7 cells influenced by eltrombopag. Mean ± SD. *n* = 3. * *p* < 0.05 vs. the LPS-treated group, *## p* < 0.01 vs. the control group, ** *p* < 0.01 vs. the LPS-treated group, ns means no significant difference.

Subsequently, we investigated the HuR-dependent mechanism of eltrombopag’s regulation of *Vegf-c*, an important factor in lymphangiogenesis in macrophages. The levels of *Vegf-c* mRNA (Figure 6A) and protein (Figure 6B,C) in LPS-activated RAW264.7 cells were all reduced by eltrombopag (*p* < 0.01). Next, we further investigated whether the down-regulation of *Vegf-c* in RAW 264.7 cells relied on HuR protein. As revealed in Figure 6D, the *Vegf-c* mRNA stability was reduced by eltrombopag. Moreover, in the RIP assay, the *Vegf-c* mRNA bound by HuR was also markedly reduced in the eltrombopag-treated group (Figure 6E, *p* < 0.05), while the non-HuR-binding mRNA *Gapdh* was not affected by eltrombopag (Figure 6E, *p* > 0.05). This evidence proves that the inhibitory effect of eltrombopag on *Vegf-c* expression in macrophage was regulated by HuR.

**Figure 7:**
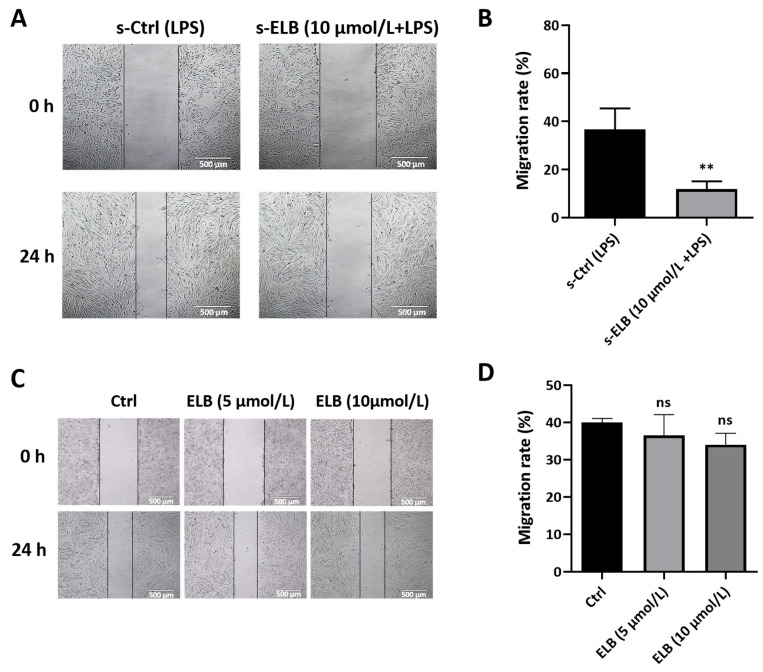
Eltrombopag regulated macrophage-mediated lymphangiogenesis. (**A**,**B**) SVEC4-10 cell migration impacted by supernatants of eltrombopag-treated macrophage, representative images (left) and quantitative analyses (right). (**C**,**D**) SVEC4-10 cell migration impacted by eltrombopag directly, representative images (left) and quantitative analyses (right). Scale bar: 500 μm. Mean ± SD. *n* = 3. ** *p* < 0.01 (*t*-test), ns means no significant difference.

It was reported that macrophages play a certain role in tumor metastasis by inducing the lymphangiogenesis factor [13]. Therefore, we explored the impact of eltrombopag on regulating lymphangiogenesis via macrophage. As indicated in Figure 7A,B, the migration rate of SVEC4-10 cells decreased notably when cultured with a culture medium derived from eltrombopag-treated macrophages (s-ELB (10 μmol/L + LPS)), as compared to those cultured with medium from non-eltrombopag-treated macrophages (s-Ctrl (LPS)) (*p* < 0.01). However, eltrombopag had no direct influence on the migration of SVEC4-10 cells (Figure 7C,D, *p* > 0.05). These data suggest that eltrombopag can inhibit SVEC4-10 cell migration through macrophage.

### 2.5. Eltrombopag Reduces Lung Metastasis of Breast Cancer In Vivo

**Figure 8:**
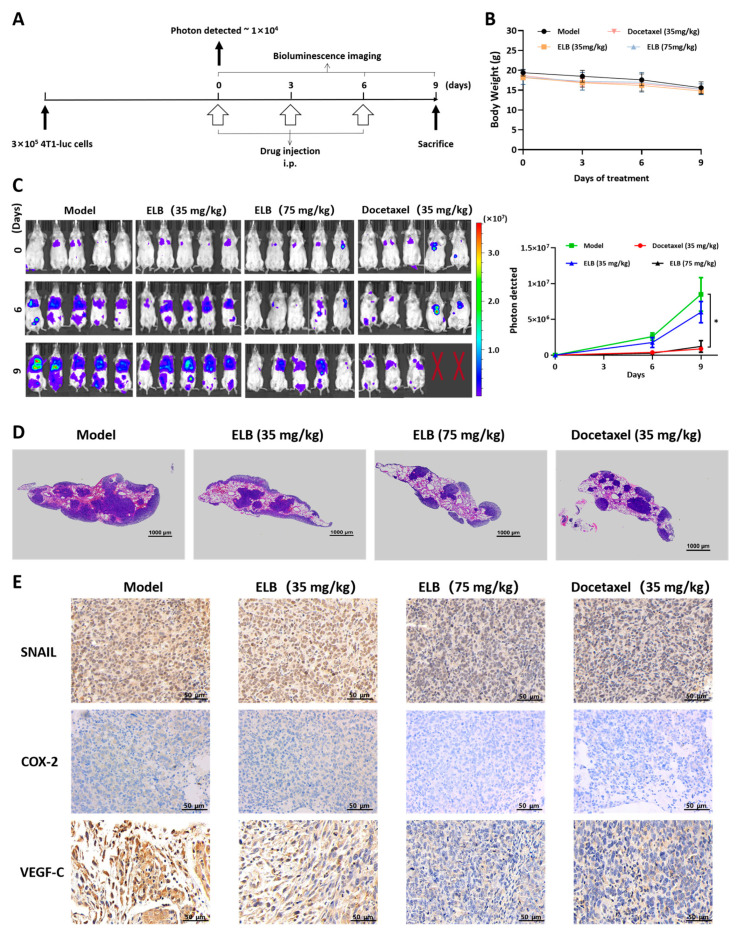
Eltrombopag suppressed lung metastasis of 4T1-Luc cells in vivo. (**A**) Schematic diagram of experimental procedures. (**B**) Body weight data of different groups after treatment. (**C**) Bioluminescent imaging data of lung metastasis after treatment with eltrombopag or docetaxel. Two mice in the docetaxel group were dead before the end of experiment, which might be due to the toxicity of docetaxel. (**D**) H&E staining of lung tissues of 4T1-Luc mice. Scale bar: 1000 μm. (**E**) SNAIL, COX-2, and VEGF-C expression in lung tissues of different groups with IHC method (Scale bar: 50 μm). Mean ± SD. *n* = 5. * *p* < 0.05 (*t*-test).

In order to evaluate the anti-metastasis ability of eltrombopag in vivo, female BALB/c mice were injected with 4T1-Luc cells via the tail vein and administrated according to the method as described in Section 4.13. The experimental scheme of this animal study is briefly shown in Figure 8A. As indicated in Figure 8B, compared to the model group, there was no obvious body weight change in the treatment group. As revealed in the bioluminescent imaging data, at day 9, compared to the model mice, the positive drug docetaxel and a 75 mg/kg dose of eltrombopag all significantly suppressed the lung photon pass values (*p* < 0.05), and a slight decline of photon flux values was detected for eltrombopag at 35 mg/kg. Moreover, eltrombopag showed a higher survival rate with the same therapeutic effect as docetaxel (Figure 8C). Sure enough, a histological examination of the lung tissue further confirmed that there were fewer metastatic nodules in the lungs of the eltrombopag (75 mg/kg) and docetaxel (35 mg/kg) groups compared to those of in the model group (Figure 8D). Moreover, we analyzed the expression of SNAIL, COX-2, and VEGF-C in the metastatic nodules of the lung tissues by IHC method. As the results show in Figure 8E, SNAIL, COX-2, and VEGF-C were all reduced in the lungs of the eltrombopag (75 mg/kg) and docetaxel (35 mg/kg) groups compared to the model group. Therefore, these data indicate that eltrombopag can play an effective therapeutic role in the lung metastasis of breast cancer.

### 2.6. Eltrombopag Suppressed Lymphangiogensis and Lymph Node Metastasis in Breast Cancer In Vivo

**Figure 9:**
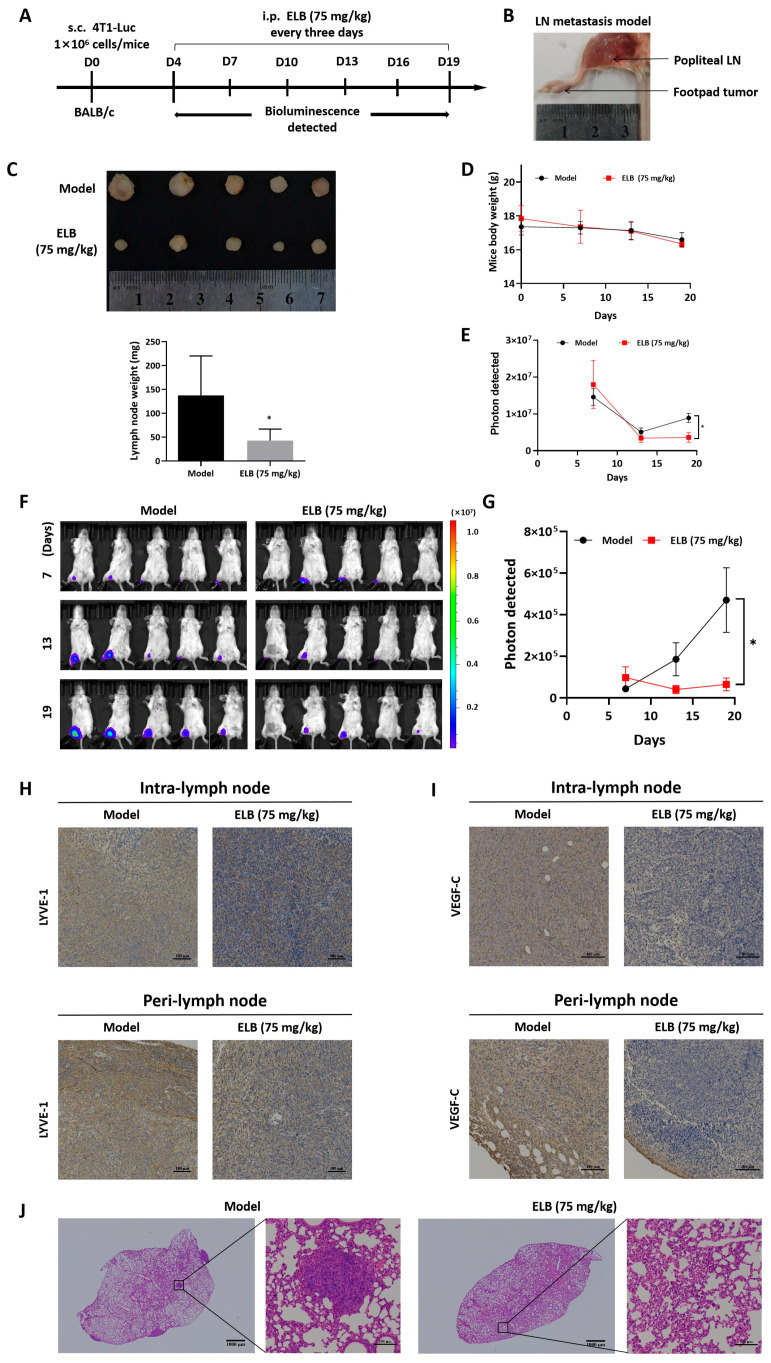
Eltrombopag suppressed lymph node metastasis of 4T1-Luc cells in vivo. (**A**) Experimental timeline of 4T1-Luc lymph node metastasis model. (**B**) Representative images of popliteal lymph node in a mouse model. (**C**) The appearance and weight of popliteal lymph node. (**D**) Body weight of tumor-bearing mice. (**E**) Bioluminescent data analysis in primary tumor site after treatment with eltrombopag. (**F**,**G**) Bioluminescent images and data analysis of popliteal lymph node metastasis after treatment with eltrombopag. (**H**) Lymphangiogenesis analysis in different sections of lymph node, lymph-vessel analysis IHC using antibodies of LYVE-1 (Scale bar: 100 μm). (**I**) Sections of popliteal lymph node in different groups, submitted to IHC using antibodies of VEGF-C (Scale bar: 100 μm). (**J**) Representative images of H&E-stained lymph node sections (Scale bar: 1000 μm). LN: lymph node. Mean ± SD. *n* = 5. * *p* < 0.05 (*t*-test).

Lymphangiogenesis and lymph node metastasis are predictors of distant metastases of tumors and are generally regarded as prognostic indicators [32]. Thus, we also investigated the anti-metastasis ability of eltrombopag in the 4T1-Luc lymph node metastasis model. The experimental scheme is shown in Figure 9A,B. First, 4T1 cells with stable luciferase expression were injected into the foot pads of BALB/c mice, and they were treated with eltrombopag every 3 days. It was observed that after treatment, a 75 mg/kg dose of eltrombopag significantly inhibited the size and weight of popliteal lymph nodes (Figure 9C, *p* < 0.05), while no dramatic body weight change was observed for a 75 mg/kg dose of eltrombopag compared to the model group (Figure 9D). As shown in Figure 9E, by IVIS detection, eltrombopag inhibited tumor growth at the primary site (*p* < 0.05). Meanwhile, tumor metastasis in the popliteal LNs was prominently decreased in the eltrombopag treatment group compared to the model group (Figure 9F,G, *p* < 0.05). LN tissues were immunostained with anti-LYVE-1 antibody and anti-VEGF-C antibody to test the microlymphatic vessels and VEGF-C positive cells. IHC with LYVE-1 confirmed that eltrombopag decreased the density of microlymphatic vessel compared to the model counterparts (Figure 9H), and the VEGF-C expression levels were significantly repressed by eltrombopag (Figure 9I). Furthermore, H&E staining showed the presence of metastatic foci in the lung tissue of the model group, but not in the eltrombopag group (Figure 9J). Collectively, all of these results suggest that, in vivo, eltrombopag exerted inhibition of tumor growth, lymphangiogenesis, and lymph node metastasis in the breast.

## 3. Discussion

HuR expression levels in tissues have been reported to be associated with a variety of pathological conditions, such as inflammation, atherosclerosis, and tumor progression [33,34,35]. In pathological conditions such as malignancies, HuR shuttles into the cytoplasm and stabilizes cancer-related genes by binding to the ARE sequence in the mRNAs. In invasive breast cancer samples, HuR is highly expressed in the cytoplasm, which is associated with a high tumor grade and poor prognosis [19,36]. In addition, the knockout of HuR reduced the aggressiveness of breast cancer cells, while the overexpression of HuR reversed this effect [37]. To date, cumulative studies have revealed that HuR inhibitors have shown an inhibitory influence on multiple HuR-targeted mRNAs, such as dehydromutactin, okicenone, and MS-444, which can reduce the expressions of *TNF-α*, *IL-2*, and *Cox-2* mRNAs [38]; quercetin and other flavonoids reduced the expressions of *TNF-α*, *c-Fos*, *IL-2*, and *Cox-2* mRNAs [39], and tanshinone and its derivatives reduced the expressions of *ERBB2*, *CTNNB1*, and *Vegf* mRNAs [40]. More recently, pyrvinium pamoate is currently in Phase I clinical trials for the treatment of pancreatic ductal adenocarcinoma in dose-escalation trials, and it has been found that its therapeutic target may be the inhibition of the nuclear cytoplasmic shuttle of HuR. Therefore, it provides a reliable experimental basis for HuR as a potential target for the treatment and diagnosis of breast cancer.

Eltrombopag is a TPO-R agonist currently used worldwide for the treatment of chronic immune thrombocytopenia (ITP) since its approval in 2008 [41,42]. Additionally, eltrombopag has also been approved for treating chemotherapy-induced thrombocytopenia (CIP) [43]. Our previous studies have found that eltrombopag could inhibit breast cancer angiogenesis and growth by targeting the HuR protein.

In the present study, we demonstrated the efficacy of eltrombopag in breast cancer metastasis in vitro and in vivo. In order to elucidate the molecular mechanisms of eltrombopag in inhibiting the metastasis of breast cancer, we investigated the effect of eltrombopag targeting the HuR protein in regulating mRNAs of tumor metastasis-related genes. First, eltrombopag was shown to disrupt the HuR-ARE interaction at the molecular level. Second, eltrombopag was found to inhibit the migration and invasion of breast cancer cells, and its inhibitory effect through HuR was verified. Third, eltrombopag suppressed macrophage-mediated lymphangiogenesis in a HuR-dependent manner. Finally, lung metastasis and lymph node metastasis of 4T1 cells was significantly inhibited by eltrombopag in vivo. Taken together, these new findings demonstrate that eltrombopag suppresses the metastasis of breast cancer cells through its action on HuR.

Tumor metastasis is a complex process involving multiple steps of the primary tumor, such as division, invasion, evasion of immune surveillance, entry into the circulatory system, and invasion of adjacent tissues. By the way, lymphangiogenesis is a critical cascade for the promotion of cancer metastasis. To date, the potential mechanisms for the regulation of HuR in breast cancer metastasis have not been fully demonstrated. On the one hand, our data suggest that the mechanism by which eltrombopag inhibits the migration and invasion of breast cancer cells may be due to the HuR-mediated reduction of *Snail*, *Cox-2*, and *Vegf-c* mRNA stability, resulting in reduced protein expression. On the other hand, eltrombopag binds to HuR in macrophages, resulting in decreased *Vegf-c* mRNA stability and protein expression, ultimately inhibiting macrophage-mediated lymphangiogenesis. Moreover, we also detected the mRNA levels of *Snail* and *Cox-2* in SVEC4-10 cells, a cell line in which the HuR protein was not elevated. Eltrombopag could not inhibit the expressions of *Snail* and *Cox-2* in SVEC4-10 cells (Figure S2), which further confirmed that eltrombopag inhibited mRNAs in an HuR-dependent manner. Consistently, the study on the effect and mechanism of eltrombopag on tumor lymphatic angiogenesis and metastasis reveals the possibility of HuR as a potential therapeutic target against tumor metastasis.

Studies have shown that HuR could regulate multiple ARE-containing mRNAs related to tumorigenesis, cancer progression, and metastasis. The present and our previous work have reported the inhibiting effect of eltrombopag on the HuR-binding mRNAs of *Snail*, *Cox-2*, *Vegf-c*, *Vegf-a* [31], and *Mmp9* [31]. However, more data about the inhibition effect of eltrombopag on other ARE-containing mRNAs are still needed, and whether *Snail*, *Cox-2*, and *Vegf-c* are critical HuR-binding mRNAs responsible for breast cancer metastasis needs further exploration.

As part of our ongoing efforts to discover and develop novel HuR inhibitors in cancer therapy, eltrombopag has emerged as a candidate that can be developed at a lower cost. However, before we can consider eltrombopag as a promising anticancer drug, more detailed studies of its anti-cancer effect and its underlying mechanism are needed. This study investigated the role of eltrombopag in regulating metastasis-related genes during breast cancer metastasis through HuR and verified the in vivo anti-metastasis activity of eltrombopag, which may accelerate the clinical application of HuR inhibitors for cancer therapy.

## 4. Materials and Methods

### 4.1. Cell Lines and Culture

The murine mammary carcinoma cell line (4T1, ATCC^®^ CRL-2539^TM^) was cultured in Roswell Park Memorial Institute (RPMI) 1640 medium (Hyclone, Logan, UT, USA), and HuR over-expression (HuROE) and silencing (ΔHuR) in 4T1 cells were generated as described previously [31]. HuR-rescued ΔHuR 4T1 cells (ΔHuR 4T1 HuROE) were generated by transient transfection of pCMV6-HuR vector with LipoHigh liposome efficient transfection reagent (Sangon Biotech, Shanghai, China); ΔHuR 4T1 cells transfected with empty pCMV6 vector were used as control (Figure S3). The murine macrophage cell line RAW264.7 was purchased from the Cell Bank of the Chinese Academy of Sciences (Shanghai, China) and cultured in Dulbecco’s Modified Eagle’s Medium (DMEM; Gibco, Waltham, MA, USA). The mouse lymphoid endothelial cell line (SVEC4-10) was purchased from BeNa Culture Collection (BNCC; Xinyang, China) and cultured in high-glucose DMEM medium (ChuanQiu Bio, Shanghai, China). Cell cultures were added with 10% fetal bovine serum (FBS; Serana, Pessin, Germany) and 1% penicillin–streptomycin (Gibco, USA). All cells were maintained at 37 °C in a humidified incubator equipped with 5% CO_2_ atmosphere.

### 4.2. Electrophoretic Mobility Shift Assay (EMSA)

An EMSA was used to detect the inhibition effect of eltrombopag on the interactions between HuR RRM12 and AREs. The ARE sequence of *Snail* mRNA (ARE*^Snail^*) was 5′-GCAAU UUAAG CAAUU UAAGC AAUUU AA-3′, the ARE sequence of *Cox-2* mRNA (ARE*^Cox-2^*) was 5′-UAUUA AUUUA AUUAU UUAAU AAUAU UUAUA UUAAA-3′, and the ARE sequence of *Vegf-c* mRNA (ARE*^Vegf-c^*) was 5′-GAUUU CUUUA AAAGA AUGAC UAUAU AAUUU AUUUC C-3′ [21,44]. ARE*^Snail^*, ARE*^Cox-2^*, and ARE*^Vegf-c^* were synthesized and labeled with 5-Carboxyfluorescein (FAM) at 5′-end by Sangon Biotech (Shanghai, China). HuR RRM12 protein was expressed in *Escherichia coli* Rosetta (DE3), and the protein purification was performed as described previously [45]. For the EMSA, the reactions included 20 nmol/L FAM-labeled AREs and 500 nmol/L HuR RRM12 protein in the presence or absence of eltrombopag. The ARE-protein complexes were separated in 1% native agarose gels with 0.5 × TBE buffer, and the electrophoretic condition was 50 V for 50 min at 4 °C [46]. The gels were scanned with a Typhoon FLA 9500 imager (GE Healthcare, Chicago, IL, USA).

### 4.3. Fluorescence Polarization (FP)

FAM-labeled AREs and HuR RRM12 protein were obtained as described in the EMSA method. In each 50 μL reactions, 20 nmol/L of 5′-FAM-AREs, 500 nmol/L of HuR RRM12 protein, and different concentrations of eltrombopag were incubated in 25 mmol/L of Tris buffer (pH 8.0) at 25 °C for 10 min. The FP values were measured with an EnVision multimode plate reader (PerkinElmer, Waltham, MA, USA). The inhibition rates and IC_50_ were calculated with GraphPad Prism 8.

### 4.4. Immunofluorescence (IF) Assays

The 4T1 cells were seeded in confocal cell culture dishes and treated with eltrombopag (5 μmol/L) or DMSO for 12 h. Then, the culture medium was removed, and cells were fixed with 4% paraformaldehyde for 20 min, permeabilized in 0.5% Triton X-100 for 10 min, and blocked in 5% BSA for 30 min. The cells were incubated with the primary antibody against E-cadherin (1:200, Cell Signaling Technology, Danvers, MA, USA) or vimentin (1:200, Cell Signaling Technology, USA) at 4 °C overnight. The cells were washed with PBS for 10 min followed by incubation with the fluorescent secondary antibody (Alexa Fluor 555, 1:1000; Shanghai, China). After washing three times with PBS, DAPI was added to the cells for 5 min. An inverted laser scanning confocal microscope (Nikon, Tokyo, Japan) was used to acquire the fluorescence images.

### 4.5. Scratch Wound Assay

A scratch wound assay was applied to assess cell motility [47]. The 4T1 cells, ΔHuR 4T1 cells, ΔHuR 4T1 HuROE cells, and SVEC4-10 cells were incubated to 100% confluence, and then cultured in serum-free medium for 12 h. Subsequently, a sterile 200 μL tip was used to generate a scratch, and then they were washed twice with PBS. For 4T1, ΔHuR 4T1, and ΔHuR 4T1 HuROE cells, eltrombopag (5 μmol/L) or vehicle was added to the plate and incubated for 24 h. Images were captured with a phase-contrast inverted microscope (N-SIM, Nikon, Tokyo, Japan) at 0 h and 24 h. To evaluate the effect of eltrombopag on the migration of SVEC4-10 cells mediated by macrophages, the SVEC4-10 cells were treated directly with eltrombopag or the supernatants of eltrombopag-treated RAW 264.7 cells for 24 h. The supernatants of the RAW264.7 cells were collected after the cells were treated with eltrombopag (10 μmol/L eltrombopag + 100 ng/mL LPS) or LPS (100 ng/mL) for 24 h. Conditioned medium containing 50% RAW264.7 supernatant was applied to the SVEC4-10 cells. The images were analyzed with ImageJ software (NIH, Bethesda, MD, USA), and the migration rate was calculated.

### 4.6. Transwell Migration and Invasion Assays

Transwell assays were used to measure the migration and invasion of 4T1 cells, ΔHuR 4T1 cells, and ΔHuR 4T1 HuROE cells. For the migration assays, 100 μL of 4T1, ΔHuR 4T1, or ΔHuR 4T1 HuROE cell suspension (1 × 10^6^ cells/mL) in serum-free medium with and without eltrombopag (5 μmol/L) were prepared and added to the upper chambers, and 600 μL medium with and without eltrombopag (5 μmol/L) was placed in the lower chambers. After incubation for 24 h, the migrated cells were fixed with 4% paraformaldehyde fix solution (PFA) at room temperature for 20 min and stained with 0.1% crystal violet (Biochannel, Nanjing, China) at room temperature for 10 min. Then, the cells were photographed with a phase-contrast microscope (N-SIM, Nikon, Japan). For counting analysis, the migrated cells were dissolved in 33% acetic acid, and the absorbance value was measured at 590 nm using a microplate reader (Thermo Scientific, Waltham, MA, USA). For the invasion assays, Matrigel (Corning, 356234, Corning, NY, USA) was used to coat the insert. Other experimental procedures were the same as those in the migration assays.

### 4.7. RNA Isolation and qRT-PCR

The qRT-PCR assays were performed to measure the expression levels of *Snail*, *Cox-2*, and *Vegf-c*. For 4T1, HuROE 4T1, and ΔHuR 4T1, the cells were treated with 1‰ DMSO or eltrombopag (5 μmol/L) for 12 h before RNA extraction. For the RAW264.7 cells, the model group was stimulated with LPS (100 ng/mL), and the eltrombopag groups were incubated with LPS (100 ng/mL) and eltrombopag (10 μmol/L or 20 μmol/L) for 12 h. The total cellular RNA was extracted using TRIzon Reagent (CWBIO, Nanjing, China) following the manufacturer’s instructions, and the RNA concentration was quantified with Nanodrop (DeNovix, USA). cDNAs were conducted using the HiFiScript gDNA Removal RT MasterMix kit (CWBIO, Nanjing, China), and quantitative PCR was performed with the ABI QuantStudio 6 Flex system (Thermo Fisher Scientific, USA) using MagicSYBR green master mix kit (CWBIO, Nanjing, China). The mRNA levels of selected genes were normalized to *β-actin* and analyzed with 2^−ΔΔCt^ method. The primers synthesized by Sangon Biotech (Shanghai, China) were as follows: *Snail* (F: 5′-CACAC GCTGC CTTGT GTCT3′ and R: 5′-GGTCA GCAAA AGCAC GGTT-3′), *Cox-2* (F: 5′-GGTGC CTGGT CTGAT GATGT ATGC-3′ and R: 5′-GGATG CTCCT GCTTG AGTAT GTCG-3′), *Vegf-c* (F: 5′-CTACA GATGT GGGGG TTGCT-3′ and R: 5′-GATTG GCAAA ACTGA TTGTG AC-3′), and *β-actin* (F: 5′-GTGGG CCGCT CTAGG CACCA A-3′ and R: 5′-TGGCT TTAGG GTTCA GGGGG-3′).

### 4.8. Western Blot

Western blot was used for protein analysis. The 4T1, HuROE 4T1, and ΔHuR 4T1 cells were treated with vehicle or eltrombopag (5 μmol/L) for 12 h. RAW264.7 cells were stimulated with LPS (100 ng/mL) and subjected to eltrombopag (10 μmol/L) or vehicle for 12 h. Subsequently, the cells were harvested using the standard protocol as described previously [48]. Antibodies against SNAIL (1:1000, 3879T, Cell Signaling Technology, USA), COX-2 (1:1000, 12282T, Cell Signaling Technology, USA), VEGF-C (1:1000, YT5297, Immunoway, Plano, TX, USA), and *β*-actin (1:1000, 3700 s, Cell Signaling Technology, USA) were incubated at 4 °C overnight. The secondary antibodies horseradish peroxidase-conjugated goat anti-mouse (Beyotime Biotechnology, A0216, Shanghai, China) and goat anti-rabbit (Beyotime Biotechnology, A0208, Shanghai, China) were incubated at room temperature for 2 h. Protein signals were detected with Meilunbio^®^ fg Super Sensitive ECL Luminescence Reagent (Dalian Meilun Biotechnology, Dalian, China) using the Azure Biosystem (Azure c600, Azure Biosystem™, Dublin, CA, USA).

### 4.9. mRNA Stability Assay

The mRNA stabilities of *Snail*, *Cox-2*, and *Vegf-c* were determined by detecting the mRNA half-life in the cells. The 4T1 and RAW264.7 cells were treated with eltrombopag as described in Section 4.7. Then, 5,6-dichlorobenzimidazole riboside (40 μmol/L) was applied to inhibit the new transcription of mRNAs, and the cells were collected at three time points (0 h, 1 h, and 2 h for 4T1 cells and 0 h, 2.5 h, and 5 h for RAW264.7 cells). The remaining mRNAs of *Snail*, *Cox-2*, and *Vegf-c* were detected with qRT-PCR. The data were analyzed with the 2^−ΔΔCT^ method, *β*-actin was employed as an internal control, and the mRNA levels at 0 h were set as 100%.

### 4.10. RNA Immunoprecipitation (RIP) Analysis

RIP assays were carried out as described with minor modifications [49]. The 4T1 cells and RAW264.7 cells were treated with eltrombopag as described in Section 4.7. Cell lysates were collected with RIPA Lysis Buffer (Sangong Biotech, Shanghai, China), and protein quantification was measured using a BCA protein assay kit (Beyotime Biotechnology, Shanghai, China). The experimental flow is shown in Figure 4D. Equal amounts of cell lysates (300 μg) were pre-bound with protein A/G beads (SC-2003, Santa Cruz Biotechnology, Dallas, TX, USA), and then HuR antibody (1:50, 12,582 s, Cell Signaling Technology, USA) was added to immunoprecipitation HuR-mRNAs complexes for 12 h at 4 °C. The mRNAs were isolated using TRIzon Reagent (CWBIO, Nanjing, China), and the cDNA was synthesized with the HiFiScript gDNA Removal RT MasterMix kit (CWBIO, Nanjing, China). The HuR-binding mRNAs (*Snail*, *Cox-2*, and *Vegf-c*) and non-HuR-binding mRNA (Gapdh) were detected with qRT-PCR and analyzed by the 2^−ΔΔCt^ method. Mouse IgG (Sepharose^®^ Bead Conjugate, 3420 S, Cell Signaling Technology) was used as a negative control. The primers used in the RIP analysis are listed in Section 4.7. The primers of *Gapdh* were 5′-AGGTC GGTGT GAACG GATTT G-3′ (F) and 5′-GGGGT CGTTG ATGGC AACA-3′ (R).

### 4.11. Luciferase Assay

The ARE sequences of *Snail*, *Cox-2*, and *Vegf-c* were synthesized and cloned into 3′-UTR of the firefly luciferase gene in the pmirGLO plasmid. The sequences of ARE*^Snail^*, ARE*^Cox-2^*, and ARE*^Vegf-c^* are shown in Figure 5I–K. The constructed plasmids pmirGLO-*Snail*-ARE, pmirGLO-*Cox-2*-ARE, or pmirGLO-*Vegf-c*-ARE were then transfected into 4T1 cells with LipoHigh transfection reagent (Sangon Biotech), and the cells were treated with 5 μmol/L eltrombopag for 24 h. The expression levels of firefly and renilla luciferase were detected with a Dual Luciferase Reporter Gene Assay Kit (Beyotime, RG027, Shanghai, China) by the EnVision Multimode Plate Reader (PerkinElmer, USA).

### 4.12. Mice

Female BALB/c mice of 6–8 weeks were purchased from the Experimental Animal Center of Shanghai University of Traditional Chinese Medicine (Shanghai, China). All the animals were raised under specific pathogen-free conditions with enough food and water before the experiments. All the procedures were carried out in accordance with the Guidelines for the Care and Use of Laboratory Animals and approved by the Animals Care and Use Committees of Shanghai University of Traditional Chinese Medicine Institutional (approval number: PZSHUTCM2100409001).

### 4.13. Metastasis Tumor Models and Treatment

Lung metastasis model: 4T1-Luc cells (3 × 10^5^ cells) were injected into female BALB/c mice via the tail vein. Lung metastasis in the mice was monitored by intraperitoneal injection with 150 mg/kg of D-luciferin potassium salt before using the IVIS^®^ Spectrum In Vivo Imaging System (IVIS; PerkinElmer, USA). Once the photon flux value for lung luciferase expression reached 10^4^ (day 0), the mice were randomly assigned to four groups (five mice in each group): vehicle saline (0.1% DMSO plus 1% tween 20), eltrombopag (35 mg/kg), eltrombopag (75 mg/kg), and docetaxel (35 mg/kg). The mice were administered intraperitoneally every three days. During the experiment, bioluminescence imaging data were acquired and quantified every three days by using Living Image software (PerkinElmer, USA). The mice were sacrificed on day 9, and the lungs were excised and fixed in 4% PFA solution for further study.

Lymph node metastasis model: 4T1-Luc cells (1 × 10^6^ cells) were subcutaneously injected into the foot pads of the mice (day 0). IVIS (PerkinElmer, USA) was applied to monitor popliteal lymph node metastasis by intraperitoneal injection with 150 mg/kg of D-luciferin potassium salt before imaging. When the image showed the bioluminescent signaling present at the site of the popliteal lymph node (day 7), the mice were randomly divided into two groups (5 animals per group): vehicle saline (0.1% DMSO plus 1% tween 20) and eltrombopag (35 mg/kg). The mice were treated with eltrombopag every three days. Animal bioluminescence signals were monitored on day 13 and day 19, and the mice were killed on day 19. The body weight of each mouse was recorded during this period. The lungs and popliteal lymph nodes were removed, fixed overnight in 4% PFA solution, and subjected to paraffin embedding.

### 4.14. Hematoxylin-Eosin Staining (H&E)

The tissues were fixed with 4% PFA and embedded with paraffin. The paraffin blocks were sliced into 5 µm slices. Subsequently, the slide was deparaffinized in xylene, rehydrated in graded ethanol, and then stained with hematoxylin-eosin routinely. The sections were photographed by fluorescence microscope systems (Nikon, Japan).

### 4.15. Immunohistochemical Staining (IHC)

Samples of the lung tissues and popliteal lymph node were applied for IHC analysis. The tissue sections were dewaxed in xylene, rehydrated in a series of graded ethanol, and subjected to antigen retrieval. The slices were incubated overnight with antibodies against SNAIL (1:1000, GB11260, Servicebio, Gent, Belgium), COX-2 (1:500, GB11077-1, Servicebio), VEGF-C (1: 100, YT5297, Immunoway, USA), or LYVE-1 (1:1000, GB113499, Servicebio), and then incubated with the secondary antibodies (HRP-conjugated goat anti-rabbit, 1:200, GB23303, Servicebio). Images were captured by using fluorescence microscope systems (Nikon, Japan) and analyzed by using the ImageJ software program.

### 4.16. Statistical Analysis

All the experimental data are exhibited as the mean ± standard deviation. GraphPad Prism 8 software was used for statistical analysis. Statistical significance was assessed by Student’s *t*-test or one-way analysis, and *p* values < 0.05 were considered statistically significant.

## 5. Conclusions

In summary, we identified eltrombopag as a metastasis suppressor in breast cancer and revealed the HuR-dependent mechanism by which eltrombopag mediates breast cancer metastasis. This study provides a basis for the new application of the old drug eltrombopag in breast cancer metastasis and highlights the value of HuR inhibitors in cancer therapy, promoting the development of novel therapies against breast cancer metastasis.

## Data Availability

Not applicable.

## Supplementary Materials

The following supporting information can be downloaded at: https://www.mdpi.com/article/10.3390/ijms24043164/s1.
