# Supplementary material for: Eltrombopag Inhibits Metastasis in Breast Carcinoma by Targeting HuR Protein
Source: Int J Mol Sci. 2023 Feb 5;24(4):3164. doi: 10.3390/ijms24043164 (PMC9963984; doi:10.3390/ijms24043164)
Supplement: Supplementary file 1 [file ijms-24-03164-s001.zip › ijms-2083913-supplementary.pdf]

Supplementary Data

Supplementary figure legends

Figure S1. IC_{50}s of ELB to 4T1 (**A**), MDA-MB-468 (**B**), and MCF-7 (**C**) cells.

Figure S2. Impact of ELB to mRNA levels of *Snail* and *Cox-2* in SVEC4-10 cells.

Figure S3. HuR protein levels in ΔHuR 4T1 cells transfected with pCMV6 vector and pCMV6-HuR vector.

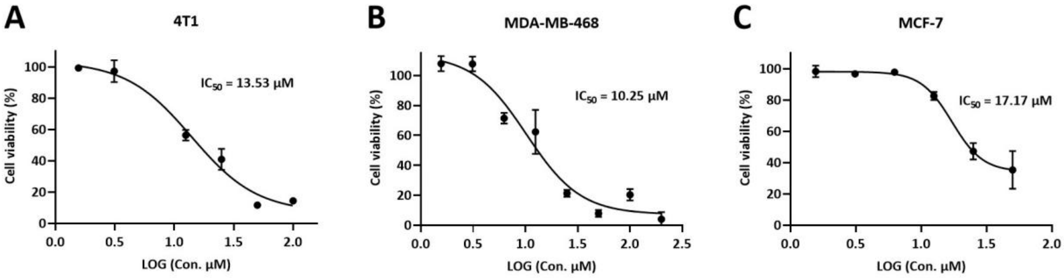

Figure S1. IC_{50}s of ELB to 4T1 (**A**) [1], MDA-MB-468 (**B**), and MCF-7 (**C**) cells.

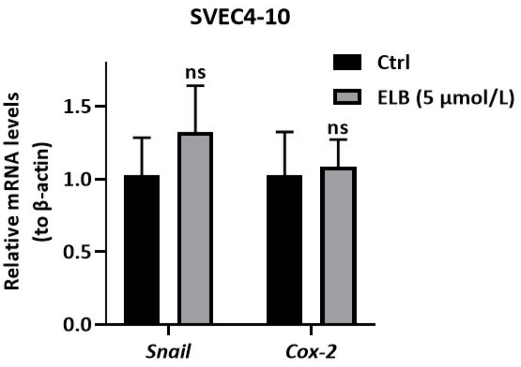

Figure S2. Impact of ELB to mRNA levels of *Snail* and *Cox-2* in SVEC4-10 cells.

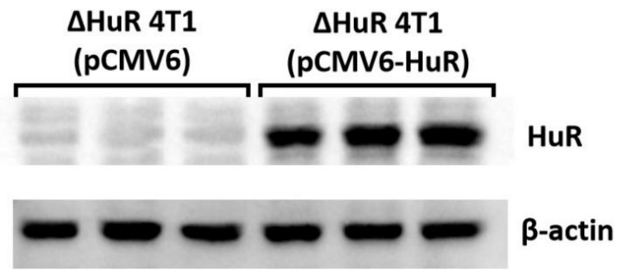

Figure S3. HuR protein levels in ΔHuR 4T1 cells transfected with pCMV6 vector and pCMV6-HuR vector.

Reference

1. Zhu, Y.; Yang, L.; Xu, J.; Yang, X.; Luan, P.; Cui, Q.; Zhang, P.; Wang, F.; Li, R.; Ding, X.; Jiang, L.; Lin, G.; Zhang, J. Discovery of the anti-angiogenesis effect of eltrombopag in breast cancer through targeting of HuR protein. *Acta pharmaceutica Sinica B.* **2020,** 10, (8), 1414-1425.
